# Editorial: The Psychology of Menopause

**DOI:** 10.3389/fgwh.2021.828676

**Published:** 2022-01-20

**Authors:** Jennifer L. Gordon, Sara Nowakowski, Caroline Gurvich

**Affiliations:** ^1^Department of Psychology, University of Regina, Regina, SK, Canada; ^2^Health Services Research, Baylor College of Medicine, Houston, TX, United States; ^3^Department of Psychiatry, Monash University, Melbourne, VIC, Australia

**Keywords:** menopause, menopause transition, mental health, depression, cognition, menopausal symptoms, intrasexual competition

The menopause transition has long been recognized as a significant time in women's lives during which the risk of mental health concerns increases. For many years, it was theorized that this increased risk was due to women's grief over the loss of their fertility (1) or that depressive mood was triggered by the “empty nest syndrome” as adult offspring moved away from the family home (2); however, these outdated theories have since been dispelled and abandoned. A biopsychosocial model has replaced these theories that emphasizes the key role of hormonal changes during the menopause transition and the direct impact these changes have on the brain (3) as well as the role they play in triggering bothersome menopausal symptoms such as hot flashes, sleep disturbances, vaginal dryness, and cognitive complaints, in turn negatively impacting quality of life (4–6).

The current Research Topic “*The Psychology of Menopause*” builds upon this more contemporary understanding of the menopause transition in a number of ways. For example, the study by Grub et al. enhances our understanding of the ovarian hormone changes that take place during the menopause transition. Specifically, in their study, Grub et al. measured ovarian and stress hormones multiple times throughout the menopause transition and made several interesting observations. First, there was large intra- and inter-individual variability in hormone levels, confirming that the longitudinal trajectory of hormonal changes occurring in the menopause transition is highly individual and largely unpredictable. Second, estradiol and progesterone levels were found to be similar across the early and late menopause transition. This novel finding raises the question of why, in light of this similarity in hormonal environment, depressive symptoms have generally been found to be more prevalent in the late relative to the early menopause transition (7). Further research is needed to investigate this issue.

Interestingly, although hormonal changes may play an important role in modulating perimenopausal mood, findings by Fiacco et al. suggest that the impact of ovarian hormones on female intrasexual competition (ISC), a term given to the rivalry between heterosexual women over men, may lessen in the postmenopausal phase. This study found that although ISC was similar among pre- and postmenopausal women, higher estradiol levels predicted more ISC in premenopausal women but not in postmenopausal women. In contrast, lower self-esteem appeared to be a more important predictor of ISC in postmenopausal women. The authors therefore suggest that ISC in the postmenopausal phase may be less motivated by reproduction and more by the desire to improve one's self-esteem by comparing oneself favorably against other women.

In line with the increasing recognition of the hormonal contributions to perimenopausal well-being, hormonal interventions are proving effective in the prevention and treatment of perimenopausal depressive symptoms (8, 9). However, recent research has also highlighted the role of psychosocial factors in amplifying the negative mental health impacts of ovarian hormone fluctuation (10), essentially pointing to a stress-diathesis model in which individuals exposed to psychosocial stressors are most vulnerable to experiencing mental illness in response to perimenopausal hormonal fluctuation. In line with this theory, mounting research suggests that, despite the importance of hormonal triggers, behavioral interventions are also effective in the prevention and treatment of perimenopausal mental health concerns as well as reducing the distress related to menopausal symptoms (7, 11–13).

Three pieces in the current issue add to this literature identifying behavioral interventions as beneficial for menopausal quality of life. First, a clinical trial by Ballantyne et al. aimed to address the cognitive concerns that are so highly prevalent in the menopause transition (14). Specifically, the feasibility study had 27 postmenopausal women undergo five 2-h weekly group cognitive remediation sessions. Though the intervention did not increase objective cognitive performance, it proved effective in increasing women's confidence in their cognitive abilities, suggesting this intervention may benefit overall quality of life in women with concerns about their cognitive health in the menopause transition.

In a similar vein, a protocol paper by Green and Furtado proposes to use a behavioral intervention to address common sexual concerns in the peri- and postmenopausal women. Indeed, sexual complaints are common in this population, with half of women reporting a decline in their interest for sex and one third reporting urogenital symptoms such as dryness, irritation, and itching (15). Specifically, the proposed trial will test the efficacy of a four-session cognitive behavioral intervention aimed at addressing sexual concerns in the peri- and postmenopausal phase.

Finally, a systematic review by Andrews et al. included in the current issue identifies and summarizes all research assessing the benefits of menopausal symptom monitoring. Though a relatively small number of studies were identified, the existing research seems to suggest that long-term monitoring of vasomotor symptoms is an effective strategy for not only improving hot flush frequency but also improved patient-doctor communication and decision making, increased health awareness, and stronger engagement in goal-setting behaviors. The findings of this review therefore provide practical recommendations for healthcare providers of mid-life women.

Taken together, these diverse reports make a valuable contribution to the current bio-psycho-social model of mental health and well-being in menopause and the menopause transition. We look forward to witnessing their research impact and the additional discoveries they spawn. In conclusion, it has been an honor to play a role in this Research Topic and we would like to thank all of the authors, reviewers, and the Frontiers staff for their help in making it a success.

## Author Contributions

JLG wrote the initial draft of the manuscript. All authors provided critical edits to the final version.

## Funding

JLG receives salary support as a Canadian Institutes of Health Research (CIHR) and Canada Research Chair. SN's research is supported by the National Institutes of Health (No. R01NR018342).

## Conflict of Interest

The authors declare that the research was conducted in the absence of any commercial or financial relationships that could be construed as a potential conflict of interest.

## Publisher's Note

All claims expressed in this article are solely those of the authors and do not necessarily represent those of their affiliated organizations, or those of the publisher, the editors and the reviewers. Any product that may be evaluated in this article, or claim that may be made by its manufacturer, is not guaranteed or endorsed by the publisher.
